# Analyzing Human Periodontal Soft Tissue Inflammation and Drug Responses In Vitro Using Epithelium-Capillary Interface On-a-Chip

**DOI:** 10.3390/bios12050345

**Published:** 2022-05-18

**Authors:** Laidi Jin, Ni Kou, Fan An, Zehang Gao, Tian Tian, Jianan Hui, Chen Chen, Guowu Ma, Hongju Mao, Huiying Liu

**Affiliations:** 1 School of Stomatology, Dalian Medical University, Dalian 116044, China; jld100302@163.com (L.J.); kouni1984@163.com (N.K.); chen1228147386@163.com (C.C.); mgw640242000@aliyun.com (G.M.); 2 Academician Laboratory of Immune and Oral Development & Regeneration, Dalian Medical University, Dalian 116044, China; 3 Center of Materials Science and Optoelectronics Engineering, University of Chinese Academy of Sciences, Beijing 100049, China; gaozh@shanghaitech.edu.cn (Z.G.); fay_tian007@hotmail.com (T.T.); huijianansouth@hotmail.com (J.H.); 4 State Key Laboratory of Transducer Technology, Shanghai Institute of Microsystem and Information Technology, Chinese Academy of Sciences, Shanghai 200050, China; 5 The Cancer Stem Cell Institute, Dalian Medical University, Dalian 116044, China; anfandlmu@163.com

**Keywords:** periodontal soft tissue, epithelium–capillary interface-on-a-chip, inflammation

## Abstract

The gingival epithelium–capillary interface is a unique feature of periodontal soft tissue, preserving periodontal tissue homeostasis and preventing microorganism and toxic substances from entering the subepithelial tissue. However, the function of the interface is disturbed in periodontitis, and mechanisms of the breakdown of the interface are incompletely understood. To address these limitations, we developed a microfluidic epithelium–capillary barrier with a thin culture membrane (10 μm) that closely mimics the in vivo gingival epithelial barrier with an immune micro-environment. To test the validity of the fabricated gingival epithelial barrier model, epithelium–capillary interface-on-a-chip was cultured with human gingival epithelial cells (HGECs) and human vascular endothelial cells (HUVEC). Their key properties were tested using optical microscope, transepithelial/transendothelial electrical resistance (TEER), and permeability assays. The clear expression of VE-cadherin revealed the tight junctions in endothelial cells. Live/dead assays indicated a high cell viability, and the astrocytic morphology of HGE cells was confirmed by F-actin immunostaining. By the third day of cell culture, TEER levels typically exceeded in co-cultures. The resultant permeability coefficients showed a significant difference between 70 kDa and 40 kDa FITC-dextran. The expression of protein intercellular cell adhesion molecule (ICAM-1) and human beta defensin-2 (HBD2) decreased when exposed to TNF-α and LPS, but recovered with the NF-κB inhibitor treatment- Pyrrolidinedithiocarbamic acid (PDTC), indicating the stability of the fabricated chip. These results demonstrate that the developed epithelium-capillary interface system is a valid model for studying periodontal soft tissue function and drug delivery.

## 1. Introduction

Periodontitis is a bacterially induced chronic inflammatory disease that compromises the integrity of the tooth-supporting tissues known as the periodontium [1]. It includes the gingiva, periodontal ligament, and alveolar bone. The key presentations of periodontitis in the early stages are gingival bleeding and recession of the gingival margin. The chronic inflammation can develop gradually into the deep periodontal tissues [2]. In severe cases, the total ulcerated area of the periodontal pocket wall can be as high as 72 cm^2^ and become a focus of infection, which provides a favorable site for material exchange between periodontal pathogenic microorganisms and their metabolites with the blood vessels. Bacteria and their metabolites invade the gingival epithelium and the underlying connective tissue, which can activate periodontal cells, promote the release of cytokines and polymorphic granulocytes, and secrete a variety of pro-inflammatory mediators. Acting as a structural barrier between the underlying tissue and the outside environment [3], the gingival epithelium provides an important contribution to the maintenance of periodontal tissue homeostasis [4,5]. It is not only a physical barrier against infection, but also actively participates in the response to infection. By interactions of epithelial cells with bacteria and metabolites, the gingival epithelium further stimulates host responses and integrates innate and acquired immunity in the innate host immune defense response [6]. Once the junction epithelial and periodontal pocket wall integrity is destroyed, the disease is complicated by tooth migration, drifting, hypermobility, and even loss, further impacting the life quality of the affected individuals [7,8]. Furthermore, considerable evidence also points to the fact that relative bacteria and their metabolites, and even the inflammatory mediators originating in the inflamed periodontium, could go beyond the oral cavity via hematogenous dissemination and thus become a risk for various systemic diseases, including atherosclerosis, adverse pregnancy outcomes, rheumatoid arthritis, aspiration pneumonia, and cancer [1,2]. Therefore, research on the gingival epithelial barrier is beneficial for establishing the ecological network in terms of periodontal tissue structure and function as well as the further exploration of host-pathogenic factors and therapeutic drugs.

In recent decades, various in vitro models have been reported to study and reproduce the gingival epithelial barrier and characterize the role of gingival epithelium cells in an immuno-inflammatory state. For example, animal models have long been the preferred method for simulating and predicting the response of periodontal tissues to drugs, pathogens, and environmental toxins. However, animal studies are costly, time-consuming, and controversial, and animal models cannot fully recapitulate the correct human physiology, as the subjects are not human [9]. Despite continuous improvements in two-dimensional cell culture technology, with their simple cell types, the cell culture assays still lack the complexity compared to the living systems. They are incapable of organ–organ or tissue–tissue communication and unable to predict complex host immune response and the effect of metabolite activity on non-target tissues [10]. Therefore, it is urgently necessary to establish more representative model systems, especially for typical human organs and diseases.

Microfluidic cell culture technology, often referred to as organ-on-a-chip, is rapidly progressing and promising in preparing in vitro models, as it could better recapitulate the key structural and functional aspects of human tissues and organs. The organ-on-a-chip systems could also enable multiple cell interactions and hence are more physiologically relevant [11]. Although most of the developed organ-on-a-chip systems cannot be considered as organs, they could partially mimic the microarchitectures of the functional units in specific organs or tissue–tissue interfaces. In addition, they could integrate various mechanical and biochemical stimuli to build valid artificial engineering organs. Usually, the organ-on-a-chip systems can be combined with biological microelectromechanical systems (bioMEMS), microfluidics, and biomimetics [10]. With the advances in microengineering technologies with microfluidic controls, these novel platforms could connect multi-organs and study their interactions on a single chip [12]. To date, the study of epithelial barrier on-a-chip that could mimic the in vivo function and microenvironment is still lacking, which hinders the understanding of the key structure of gingival human periodontal soft tissue.

From the perspective of organ-on-a-chip construction, the oral cavity is a very special organ that is an open system that is constantly challenged by microorganisms and their toxic products and antigenic components. At the same time, the oral cavity is a complex micro-ecological environment, and a balanced relationship between host and microbes is a prerequisite for periodontal health. The effectiveness of a model depends on its ability to reproduce the key physiological and biological characteristics of its in vivo prototype. The key features of the periodontal soft tissue barrier include: (1) the first barrier, composed of tight junctions between epithelial cells, protecting deep tissues against invasion by foreign microorganisms; (2) co-culture of epithelial and endothelial cells, including material exchange and indirect cell contact, which plays an important role in regulating barrier function via cell–cell signaling; (3) the selective permeation of macromolecules, the regulatory function on the proteins expression in inflammatory states, and the maintenance of high resistive resistance values representing integrity and effectiveness of the structure.

This study discusses and provides procedures for developing epithelium–capillary interfaces on a chip, including the fabrication of bilayer polymers, cell culturing procedure, validation of the developed models through optical imaging, transient permeability tests, and the proteins of intercellular cell adhesion molecule (ICAM-1) and human beta defensin-2 (HBD2) measurement under inflammation with or without inhibitor. Overall, we demonstrate a realistic microfluidic platform, comprising the smallest epithelium–capillary interface on-a-chip, which is capable of mechanically and biochemically modulating the barrier function.

## 2. Materials and Methods

### 2.1. Isolation and Culture of Cells

For human studies, approval from the Ethics Committee of Dalian Medical University as well as written informed consent from all participants was obtained. The study was performed in accordance with the principles of the Declaration of Helsinki. As previously reported [13], with the informed consent of the patients, 1 to 2 mm of healthy gingival tissues were surgically dissected under aseptic conditions from patients aged 18 to 30 years who needed to have their wisdom teeth extracted due to wisdom tooth obstruction. After repeated rinsing in phosphate-buffered saline (PBS) without calcium and magnesium containing streptomycin-penicillin and fungicides, gingival tissues were incubated with 0.4% dispase at 4 °C for 16–18 h. The epithelium was separated from the lamina propria and trimmed into 0.3 cm^2^ pieces, trypsinized for 10–15 min at 37 °C in 0.05%/0.53 mM trypsin EDTA, pipetted for 5–8 min, and the supernatant was removed by centrifugation at 700 rpm for 5 min. Next, the cell pellet and tissue fragments were collected and resuspended in keratinocyte growth medium (HuMedia-KG2, Kurabo, Osaka, Japan, KK-2150S). The cells were cultured at 37 °C with 5% CO_2_ with the first 3-day fluid change and then every other day to observe the growth status of the cells and used for assays between 4 and 6 passages. Human vascular endothelial cells (Lonza, C2517A) were cultured in EBM-2 medium (Lonza, Japan). All cell culture-related reagents were purchased from Life Technologies Corporation, unless otherwise noted.

### 2.2. Primary Cell Identification

AE1/AE3 antibody was used to specifically characterize gingival epithelial cells. The digested gingival epithelial cells were inoculated on glass slides, washed with phosphate buffered saline (PBS) three times after culturing for 24 h, fixed with 4% (volume fraction) formaldehyde and incubated for 15 min at 25 °C, and 0.1% (volume fraction) polyethylene glycol octyl phenyl ether (Triton X-100) was also added for an incubation time of 15 min at 25 °C in order to penetrate cellular membranes. For treatment, 3% (volume fraction) hydrogen peroxide (H_2_O_2_) was also added. After the serum was blocked, mouse anti-human keratin monoclonal antibody AE1/AE3 (Beijing Zhongshan Jinqiao, China) was added overnight at 4 °C, rinsed with PBS, and then biotin-labeled secondary antibody (diluted by volume ratio 1:50) and horseradish enzyme were added in sequence. The labeled streptavidin was incubated at 25 °C for 30 min each, DAB developed, observed under a microscope, counterstained with hematoxylin, and mounted.

### 2.3. Measurement of the Transepithelial/Transendothelial Electrical Resistance

TEER was measured using 12 mm transwell inserts with 8 µm pore size polycarbonate membrane (Dow Corning, Midland, MI, USA). The values of TEER were obtained by transferring the transwell inserts into an Endohm chamber. Concentric counter electrodes above and below the membrane caused overlapping current densities to flow through the membrane, and EVOM2 (WPI, Sarasota, FL, USA) calculated the transmembrane resistance based on the currents. All TEER values were obtained after subtracting the background and time of insertion into the membrane [14].

**Figure 1:**
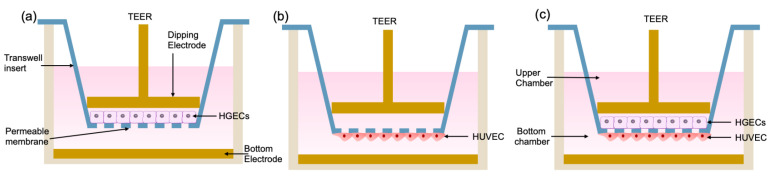
Schematic description of transepithelial/transendothelial electrical resistance assays. (**a**) HGECs are seeded onto the upper non-coated porous membrane of the transwell; (**b**) HUVEC are seeded onto the bottom non-coated porous membrane of the transwell; (**c**) HGECs and HUVEC are respectively cultured on the upper and bottom sides of the porous membrane within the transwell.

As shown in Figure 1, we divided the test into three groups. The first group was inoculated with HGEC cells in the upper layer of the chamber, and the lower layer was blank (Figure 1a); the second group was inoculated with HUVEC cells in the lower layer of the chamber, and the upper layer was blank (Figure 1b). The third group was a co-culture chamber in which HGEC cells were inoculated in the upper layer, and HUVEC cells were inoculated in the lower layer (Figure 1c).

### 2.4. Device Assembly and Operation

The upper and lower layers of the microfluidic device were produced using SU8–3050 negative photoresist (Dow Corning, Midland, MI, USA) and polydimethylsiloxane (PDMS, Dow Corning, Midland, MI, USA) according to the standard soft lithography and microfabrication methods [15]. The channels of the two layers of the microfluidic device were separated by a thin (10 μm) semi porous polyester membrane (1 μm pores) that was purchased from Sterlitech Corporation (Auburn, WA, USA). The bonding of the device was carried out as previously described [16]. PDMS and porous polyester (PETE) membranes were immersed for 20 min in a 1% (volume fraction) aqueous solution of 3-Glycidoxypropyltrimethoxysilane (GLMYO, Sigma-Aldrich, St. Louis, MO, USA) and a 5% (volume fraction) aqueous solution of 3-Aminopropyltriethoxysilane (APTS, Sigma-Aldrich, St. Louis, MO, USA), respectively, rinsed in water, and dried in a stream of compressed air. The porous PETE membrane was finally aligned and brought into contact with the PDMS layers comprising the basal microfluidic compartment that formed the microfluidic compartment and then the surfaces were pressed together. A porous PETE membrane was sandwiched between the two microfluidic channels during bonding. The assembled microfluidic chips were finally baked at 60 °C overnight.

### 2.5. In Vitro Co-Culture Epithelium–Capillary Interface Models and Assembly

To form a bilayer epithelial-capillary on the chip, the epithelial and endothelial cell suspensions were seeded on the upper and lower sides of the porous membrane in the device, respectively. Prior to cell inoculation, the chambers of the sterilized chip were filled with liquid and immersed in culture medium overnight. First, the chip was turned over so that its lower chamber could face upwards, and HUVEC cells were introduced into the lower chamber with a pipette at a concentration of 1 × 10^5^ cells/mL. After resting for 2 h for cells to attach, the chip was turned over again so that its upper chamber faced upward and HGEC cells were introduced into the upper chamber at a concentration of 8.9 × 10^4^ cells/mL. After HGEC cells adherence, the cell culture medium was refreshed every 24 h with the HGEC cells side facing up. The cells in both microchambers were grown to confluence within three days. Once the cells reached confluence, they were treated with culture medium containing either LPS (10 μg mL^−1^) or TNF-α (10 ng mL^−1^) to promote the formation of inflammation model in vitro. Inflammation stimulation experience and all cell cultivation was carried out in the incubator, which maintained a constant interior environment at 5% CO_2_ and 37 °C.

### 2.6. Cell Staining

For morphological observations, light-phase and fluorescence imaging were used. Live/dead experiments were used to detect the cellular activity, and immunostaining was used to observe the expression of F-actin and tight junction marker proteins VE-cadherin. In order to analyze the viability of cultured cells, calcein AM and ethidium homodimer-1 of the Live/dead Kit (Invitrogen, Carlsbad, CA, USA) were mixed in a ratio of 1:4, incubated for 15 min and imaged using a fluorescence microscope. Immunostaining for both types of cells was fixed with 4% (volume fraction) formaldehyde and incubated for 10 min at 25 °C, and 0.1% Triton X-100 (volume fraction) was also added for an incubation time of 15 min at 25 °C in order to penetrate cellular membranes. For treatment, 3% (volume fraction) bovine serum (Invitrogen, Carlsbad, CA, USA) in a permeabilization buffer was also added. After the serum was blocked, the primary antibody was added in a blocking buffer overnight at 4 °C, and then a secondary antibody or a compatible counterstain for the cytoskeleton was added in sequence. The labeled streptavidin or a compatible counterstain for the cytoskeleton was incubated at 25 °C for 1 h, and then the nucleus was stained with DAPI (Sigma-Aldrich, St. Louis, MO, USA) for 5 min at 25 °C, protected from light. Rabbit anti-VE-cadherin (Invitrogen, Carlsbad, CA, USA) at 1/25 dilution was used in conjunction with Alexa Fluor 488 goat anti-rabbit secondary antibody (Invitrogen, Carlsbad, CA, USA) for HUVEC cells. Phalloidin- iFluor^®^594 (Abcam, Cambridge, UK) at a 1/1000 dilution in PBS was used for HGECs. Images was taken using a Nikon fluorescence microscope.

### 2.7. Characterization of Cell Layers

To assess barrier permeabilities to large compounds, fluxes of fluorescent tracers over a wide range of sizes were measured after steady-state HUVEC layers had been reached. The absorption and barrier capacities of the HUVEC layers were evaluated by measuring the apparent permeability (P_app_) of labeled dextran (FITC-dextran, MW 40 kDa, and MW 70 kDa, Sigma-Aldrich, St. Louis, MO, USA) through the cell layers. A Horiba FluoroMax-4 spectrofluorometer and Orient KOJI Semi-Micro spectrofluorometer cuvettes were utilized to perform emission fluorescence measurements. Slit sizes were set at 1.5 nm for all monochromators. All processes were conducted at room temperature (25 ± 1 °C). According to the manufacturer’s requirements, we prepared a concentration gradient of FITC-dextran standard sample, measured the fluorescence intensity value at 518 nm, and made a standard curve to calculate the concentration of the sample to be tested; P_app_ was calculated using the equation:

$$P_{\mathrm{app}}\left[\mathrm{cm}/s\right]=\Delta C/\left(A\times C_{0}\times \Delta t\right)$$

where A = area of mass transfer, C_0_ = donor concentration of reagent in upper medium, and ∆C/∆t = transmembrane transportation rate.

All permeability assays were conducted after day 3 of endothelial culture.

### 2.8. Analysis of the Protein Expression

The effluent of the medium was analyzed for a panel of intercellular cell adhesion molecule (ICAM-1) and human beta defensin-2 (HBD2) using custom ELISA assay kits (Abbkine, Wuhan, Hubei, CN). Analyte concentrations were determined according to the manufacturer’s instructions using enzyme-labeled instrument coupled with Origin software (OriginLab, Northampton, MA, USA). For inflammation stimulation experiments, healthy epithelia were treated with LPS (10 μg mL^−1^) or TNF-α (10 ng mL^−1^) for 24 h, and amounts of secreted ICAM-1 and HBD2 were measured 24 h after treatment. For periodontitis drug studies in chips containing cocultures of human gingival epithelium and endothelium, the endothelial cells were treated with 25 mM NF-κB inhibitor PDTC (Ammonium pyrrolidinedithiocarbamate, Sigma-Aldrich, St. Louis, MO, USA) or blank medium under for 4 h before LPS (10 μg mL^−1^) or TNF-α (10 ng mL^−1^) was delivered into the epithelium channel for 24 h. The vascular effluents and epithelium effluents were then collected for ICAM-1 and HBD2 analysis.

### 2.9. Statistical Analysis

All results and error bars are presented as mean and SEM. Data were analyzed with an unpaired Student’s *t*-test using Origin (OriginLab, Northampton, MA, USA) or Excel software (Microsoft, Redmond, WA, USA). Differences between groups were considered statistically significant when * *p* < 0.05, ** *p* < 0.01, *** *p* < 0.001.

## 3. Results

### 3.1. Cell Identification

**Figure 2:**
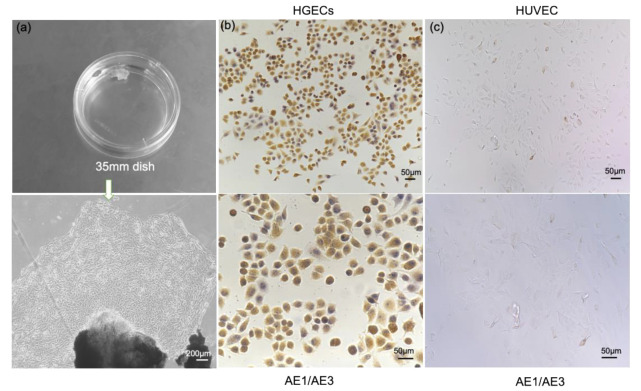
Primary gingival epithelial cells extraction and indirect immunocytochemical staining of cells with cytokeratin staining. (**a**) Human gingival tissue mass and the first passage HGECs showing slabstone-shaped; (**b**) HGECs immunohistochemical analysis with keratin antibody showing a positive result (the cytoplasm is brownish yellow, nuclear staining is negative, and it is blue after counterstaining with hematoxylin); (**c**) HUVEC as the control group, the expression of cytokeratin is negative.

Mouse anti-human keratin monoclonal antibody AE1/AE3 helped in the identification of primary gingival cells. As shown in Figure 2a, epithelial cells appeared from the edge of the tissue block and gradually expanded around the tissue block on the third day after culture. The cells were polygonal in shape, tightly inlaid, and arranged like paving stones. The nucleus was large, round or oval, with several nucleoli, the cell size was relatively uniform, the cytoplasm was plump, and mitotic cells were seen in each phase. Immunocytochemical staining results shown that the mouse anti-human keratin monoclonal antibody AE1/AE3 stained positively, as shown in Figure 2b; the cytoplasm was brownish yellow, nuclear staining was negative, and it was blue after counterstaining with hematoxylin. The control group was negative in Figure 2c, indicating that the primary cultured cells were HGECs.

### 3.2. TEER Measurements in Transwell

**Figure 3:**
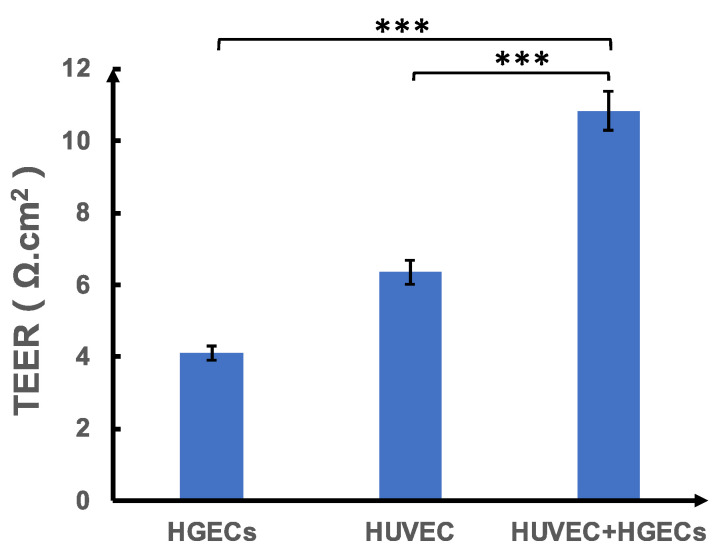
TEER-measurements in HGECs, HUVEC cells, and co-culture of HGECs and HUVEC cells. Results are mean ± SD from three or more experiments and data are analyzed by two tailed student’s *t*-test *** *p* < 0.001 compared with the monolayer.

The inoculation method of the cells used in the study and the co-culture method of the two cell types are the key factors to assess the success of the model, as is the ability to maintain the normal barrier function of each cell layer to a certain extent. Transepithelial/transendothelial electrical resistance (TEER) is a widely accepted quantitative technique to measure the integrity of tight junction dynamics in cell culture models of endothelial and epithelial monolayers [17]. Figure 3 shows the graphs of the TEER of transwell inoculated with HGECs, HUVEC cells, and HGECs and HUVEC cells simultaneously. It can be seen that the TEER values of the two cells in co-culture are higher than those of the two cells in separate cultures, indicating that the cell co-culture method used in this study can maintain the normal barrier function of the two cells to a degree.

### 3.3. Design and Construction of Periodontal Soft Tissues on Chip

**Figure 4:**
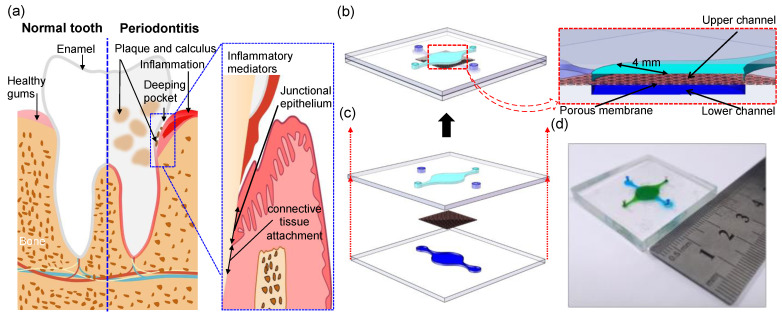
Structure and design of the developed epithelium–capillary interface-on-a-chip. (**a**) Victory anatomy of periodontal tissue; (**b**) the fully fabricated periodontal soft tissue chip, the system comprises two perpendicular flow channels: channel heights are 200 μm, and chambers radium are 4 mm (lumen and albumen); (**c**) the chip consists of two PDMS layers, and a piece of polycarbonate membrane; (**d**) close-up view. Channels model the lumenal (green) and ablumenal (blue) sides of the epithelium unit. HGEC and HUVEC cells are cultured on the lumenal and ablumenal sides of the enclosed porous membrane, respectively.

To construct the epithelium–capillary interface of the periodontal soft tissue, we used soft lithography to create a microfluidic device made of PDMS containing an upper circular chamber with a radius of 4 mm separated from a parallel lower micro-vascular chamber (radius of 4 mm) by a thin (10 μm), porous (1 μm pores) PETE membrane in Figure 4. Figure 4a shows that in a healthy state of periodontal support tissue, the gingiva is pink in color, thin, and tightly wrapped around the cervical part of the tooth and close to the tooth surface, and the underlying junctional epithelium is connected to the tooth surface, which is well-sealed. The soft-hard interface plays a key role in protecting the health of periodontal support tissues. As shown in Figure 4b, we used porous membrane as the cell attachment medium, and inoculated HGEC cells on the lumenal side of the porous membrane to form the epithelial barrier and HUVEC cells on the ablumenal side to simulate the periodontal microvascular barrier. The porous structure of the porous membrane can recognize the intercellular material exchange and signal transmission so that we can successfully simulate the functional unit of the periodontal soft tissue epithelium–capillary interface. When subjected to long-term stimulation by plaque microorganisms and their products in the cervical and gingival grooves, the gingival epithelium, as the first line of defense against microorganisms, will first lead to gingival inflammation and may further activate the vascular endothelial cells in the dense vascular plexus below it that runs parallel to the tooth surface, causing the body’s immune response and secreting pro-inflammatory factors that will accelerate the destruction of deep periodontal tissues in Figure 4b.

### 3.4. Reconstitution of a Functional Epithelium–Capillary Interface

**Figure 5:**
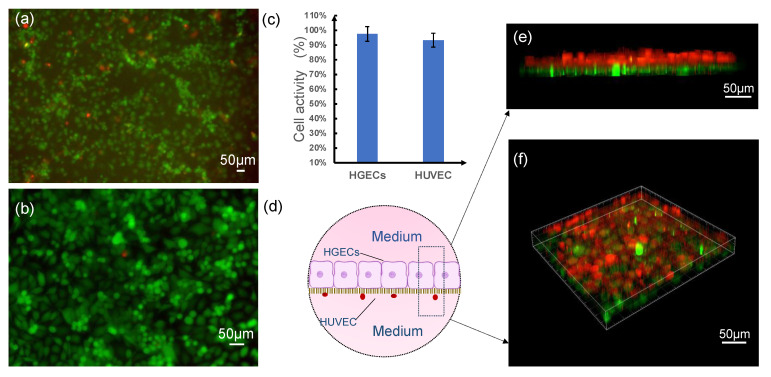
On-chip formation of an epithelium-capillary interface. (**a**,**b**) Live/dead stain (green: live, red: dead) of HUVEC and HGEC cells on day 3 of culture on the porous membranes. (**c**) Statistical analysis of cell viability. (**d**) Three-dimensional schematic diagram and (**e**,**f**) reconstructed views of interface formed by HUVEC and HGECs cells on the top PETE membrane tracked by celltracker^TM^ orange (HGECs) and celltracker^TM^ green (HUVEC).

The viability and co-cultivation status of the two kinds of cells in the chip are an important basis for the successful construction of the microdevice. Imaging results were indicative of in vivo like morphologies for both cell types, validating structural requirements for the epithelium–capillary interface. As shown in Figure 5a,b, results from live/dead assays conducted on day 3 of endothelial and epithelial culture on membranes indicated high cell viability (>90%) of endothelial cells cultured in the system (Figure 5c). Similar cell survival was seen for epithelial cells cultured in the system. The celltracker^TM^ (orange) and celltracker^TM^ (green), which represent HGECs and HUVEC cells, were stained on the upper and lower surfaces of the top membrane, respectively, simulating specific space layout of the physiological conditions (Figure 5d). It can be observed in the confocal immunofluorescence microscopy analysis (Figure 5e,f) that the HUVEC on the upper surface maintained tight connections, whereas the HGECs on the lower surface were distributed sparsely to maintain their proportions and were similar in physiology.

### 3.5. Biological Characterization of Cells in the Chip Device

**Figure 6:**
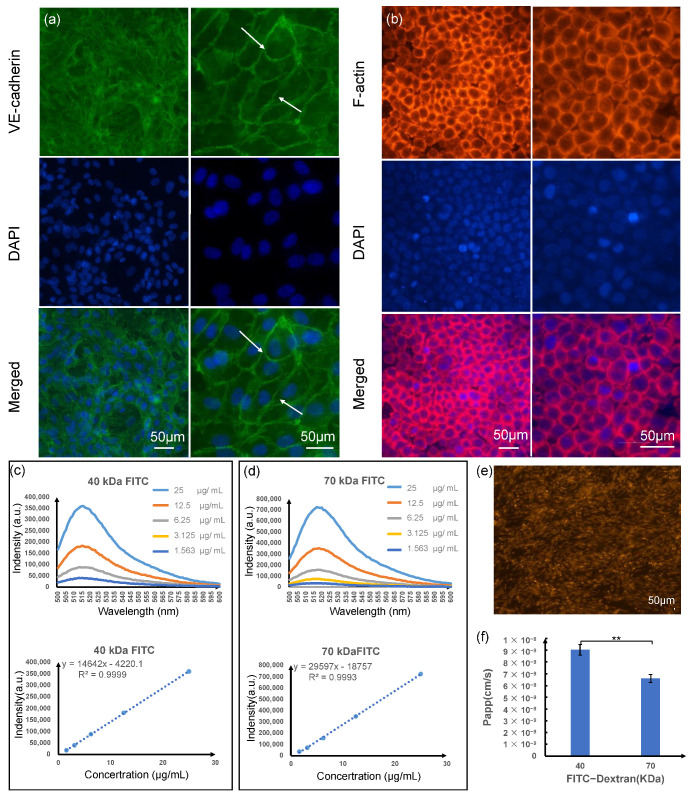
Biological characterization of the two types of epithelium−capillary interface cells on chip. (**a**) Representative images of the expression of tight junction protein VE-cadherin (green) and DAPI (blue) of HUVEC; (**b**) the cytoskeleton of HGECs on membrane (red; stained with F−actin) are visualized with nucleus (blue; stained with DAPI); (**c**) the emission spectra and fluorescence intensity (emission at 518 nm) of different concentration standards of the 40 kDa FITC−dextran; (**d**) the emission spectra and fluorescence intensity (emission at 518 nm) of different concentration standards of the 70 kDa FITC−dextran; (**e**) the fluorescence image of the HUVEC cells layer (stained with celltracker^TM^ orange) on the porous membrane; (**f**) the apparent permeability (P_app_) of soluble reagents with 40 kDa and 70 kDa molecular weight FITC−dextran through the HUVEC cells layer, *n*  =  3. Error bars represent the standard error of the mean (SEM) of three independent experiments. Two-tailed significance was set to ** *p*  <  0.01.

Each type of cell had unique roles and interactions with each other when periodontal soft tissue performed their functions. To ensure that each type of cell kept functions well during the periodontal soft tissue on-chip device operation, several biomarkers of cells on the chip were characterized. The HUVEC cells mainly performed the function as a barrier, selectively allowing substances to pass through. As shown in Figure 6a, the location and intensity of VE-cadherin staining illustrated that a tight junction mono-dermic structure emerged. There are pictures collected under different excitation lights. The green represents the VE-cadherin protein expression image, the blue DAPI represents the location of the cell nucleus, and the part indicated by the white arrow represents the formation of tight junctions between cells. Similarly, as shown in Figure 6b, the cytoskeleton (F-actin) showed that the HGECs were in good condition as the cells were dispersed from one another and stable cell numbers were maintained. The red color depicts cytoskeleton (F-actin) protein expression image, and the blue DAPI represents the location of the cell nucleus.

To reproduce endothelial barrier function, the HUVEC cell layer was generated in the microdevice. We cultured the HUVEC cells on the PETE membrane for 48–72 h to form tight junctions between HUVECs. We then tested the barrier capability of the HUVEC layer by measuring the apparent permeability of 40 kDa dextran and 70 kDa dextran. Figure 6c–f shows the apparent permeability (P_app_) of soluble reagents with different molecular weights through the HUVEC layer. Figure 6c shows the fluorescence intensity (emission at 518 nm) and the emission spectra of different concentration standards of the 40 kDa FITC-dextran. In addition, Figure 6d shows the fluorescence intensity (emission at 518 nm) and the emission spectra of different concentration standards of the 70 kDa FITC-dextran. Figure 6e shows the fluorescence image of the HUVEC cell layer (stained with celltracker^TM^ orange) on the porous membrane. Figure 6f shows that the the P_app_ of 40 kDa FITC-dextran was much higher than that of 70 kDa FITC-dextran, and that both values are below 10^−7^ cm/s, indicating that the vascular endothelial cell layer formed within the chip was molecularly selectively permeable and consistent with capillary properties.

### 3.6. Periodontal Soft Tissue Inflammation and Evaluation of Therapeutic Responses On-Chip

**Figure 7:**
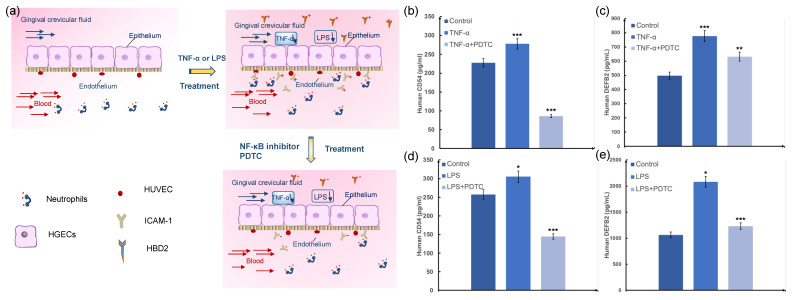
Periodontal soft tissue inflammation and therapeutic responses on-chip. (**a**) Schematic diagram of inflammation model and drug model; (**b**,**c**) the graphs show effects on production of the human CD54 (ICAM-1), Human DEFB2 (HBD2) stimulation with TNF-α (10 ng mL^−1^) in the absence or presence of medicine on the epithelium-capillary interface chip; (**d**,**e**) The graphs show effects on production of the ICAM-1, HBD2 stimulation with LPS (10 μg mL^−1^) in the absence or presence of PDTC on the epithelium–capillary interface chip. *n*  =  3. Error bars represent the standard error of the mean (SEM) of three independent experiments. Two-tailed significance was set to * *p*  <  0.05 and ** *p*  <  0.01, *** *p* < 0.001.

We then explored whether this microdevice could reproduce the complex organ-level cascade of responses in human periodontal tissue. As Figure 7a shows, periodontal inflammation is a multi-step cascade involving a highly synergistic response with the production and release of early response cytokines by epithelial cells, the activation of vascular endothelium by upregulated leukocyte adhesion molecules such as intercellular adhesion molecule-1 (ICAM-1), promoting the adhesion of leukocytes in the periodontal microcirculation and their migration to the epithelium, which in turn leads to a series of inflammatory responses. The expression of human beta defensin (HBDs), an important part of oral natural immunity, causes diseases in the periodontium [18,19,20]. We selected the most common inflammatory factor LPS and cytokine TNF-α as initiators of the in vitro periodontal inflammation model and the NF-κB inhibitor PDTC as a drug evaluation model to construct an in vitro periodontal soft tissue inflammation drug evaluation system [21,22], and then detected the changes in ICAM-1 and HBD2 biomarkers related to inflammation.

As shown in Figure 7b,c, the expressions of ICAM-1 and HBD2 increased after 10 ng mL^−1^ TNF-α and 10 μg mL^−1^ LPS treated compared with the untreated control group and is statistically significant. Compared with the TNF-α or LPS stimulated group without pretreatment with the drug PDTC, the expression of HBD2 and ICAM-1 showed a statistically significant reduction. Similarly, the expression of ICAM-1 and HBD2 increased after 10 ng mL^−1^ LPS stimulation compared with the untreated control group and is significant, as shown in Figure 7d,e. In comparison with the LPS-stimulated group without pretreatment with the drug PDTC, the expression of HBD2 and ICAM-1 showed a statistically significant reduction.

## 4. Discussion

The host–microorganism homeostasis of the epithelial barrier within periodontal soft tissue determines how these key cells function and interact with their surrounding environment [5]. In order to capture the abnormalities of the periodontitis and better replicate epithelium behavior than monolayer cultured cells in the host immune response, co-culturing of main cells such as epithelial cells and incorporation of a defined perfusable vasculature can help investigate the evolving epithelial barrier and its effect on drug bioavailability inside the vascularized periodontal soft tissue.

In this study, a highly integrated periodontal soft tissue chip model was constructed based on the anatomical structure and physiological functions of periodontal soft tissues. Cell spatial arrangement and distribution ratio are used to study periodontal soft tissue physiology and cell biological behavior under inflammatory conditions. The characteristic of this microfluid model is that it used many bionic designs, making the established in vitro model more compatible with in vivo physiology. Physiologically, epithelial tissue acts as the first barrier against foreign invasions. It plays an important role in controlling microbial infections, protecting subepithelial tissues, and maintaining periodontal tissue homeostasis [23]. The cytoskeleton (F-actin) showed a good condition of HGEC cells as the cells were dispersed from one another and kept the stable cell numbers, ensuring the physical barrier function of the epithelial barrier. In addition, the gingival epithelium not only functions as a physical barrier, but it also produces antibacterial substances such as defensins. When periodontal tissue was invaded by microorganisms, the inflammatory mediators produced by epithelial cells also activated endothelial cells to enhance the expression of endothelial cell adhesion molecules, promoted the release of cytokines and polymorphonuclear granulocytes, and secreted a series of pro-inflammatory mediators that can cause periodontal tissue microvascular and tissue disease. This results in monocytes/macrophages infiltrating the blood vessel wall, causing damage to other organs caused by small vessel disease. Therefore, the barrier function of vascular endothelial cells cannot be ignored in the periodontal soft tissue barrier [23,24]. Here, the HUVEC cell monolayer can readily express tight junctions and higher permeability to tracers of lower stokes radius, indicating that smaller compounds pass through junctions more easily and maintain an effective endothelial barrier. In addition, endothelial and epithelial culture on the membranes indicated high cell viability (>90%) of HUVEC cells and HGEC cells cultured in the microdevice on day 3, the much thinner culture membrane, decreasing the distance between co-cultured cells from the epithelium-capillary interface model, and this made HGEs and HUVEC cells maintain a small population while mimicking the function of the gingival epithelial barrier. Overall, imaging indicated that the microdevice exhibited characteristics desirable for gingival epithelial barrier study, and cells are co-cultured in close contact with significantly lower costs and timescales than in vivo studies.

Compared with animal models and traditional flat-panel models, different types of functional cells and perfusion media can be easily and independently recovered from this device. Therefore, multiple biomarkers of each type of cell can be readily characterized by an off-line analysis, such as immunofluorescence staining. In the present study, we used this model to examine interactions between HGECs and HUVEC cells in periodontitis. We observed that both LPS and TNF-α could increase the expression with ICAM-1 and HBD2, the key factors in the activation of endothelial function and oral natural immunity. The adding of inhibitor PDTC can decrease in the chip and is consistent with the expression in animal experience, and this cell-parallel, controlled and repeated environment is not available with tradition models. Thus, the epithelium–capillary interface-on-a-chip may be a relevant model with making a dynamic microenvironment providing shear stress stimulation to the cells and allows the improved analysis of test compounds and controlled delivery compared to static models for periodontal soft tissue. However, it can also be adapted for studies of inflammation-based systemic disease such as meningitis, coronary heart disease, Alzheimer’s disease, and leukemia.

Limitations of this study include the use of HUVEC cells instead of periodontal endothelial cells derived from the periodontal microvasculature. We expect the effects on related medicine and cytokine secretion to be even more pronounced once periodontal associated microvasculature cells are used. Importantly, this work reproduced the key function interface of periodontal soft tissue and proposed the application of organ-on-a-chip in the oral cavity, which provides a novel in vitro platform for other oral diseases and other related organ diseases.

## 5. Conclusions

In conclusion, we proposed an epithelium–capillary interface on-a-chip device for studying periodontal soft tissue inflammation. The proportions and spatial structures of HUVEC and HGEC cells were sequentially integrated to mimic the anatomy and microenvironment of periodontal soft tissues in vivo. Our results showed that this novel periodontal soft tissue device was able to reproduce the inflammatory process induced by LPS or TNF-α in major periodontal soft tissue cell lines while measuring multiple biomarkers of each periodontal soft tissue cell line to understand the intercellular communication between one another. Thus, this in vitro epithelium–capillary interface microarray device could serve as a potential platform for drug-induced periodontal soft tissue function and disease.
